# Strengthening routine immunization through measles-rubella elimination

**DOI:** 10.1016/j.vaccine.2018.07.029

**Published:** 2018-09-05

**Authors:** Robin J. Biellik, Walter A. Orenstein

**Affiliations:** a Geneva, Switzerland; b Emory Vaccine Center, Emory University School of Medicine, Atlanta, USA

**Keywords:** Measles elimination, Rubella elimination, Vaccine-preventable disease, Vaccination coverage, Routine immunization, Supplementary immunization activities

## Abstract

The 2016 mid-term review of the Global Measles-Rubella Strategic Plan 2012–20 for achieving measles-rubella elimination concluded that the full potential of strategies and activities to strengthen routine immunization (RI) service delivery had not been met. In December 2017, we contacted WHO and partner agency immunization staff in all six WHO Regions who identified 23 countries working on measles or rubella elimination that have implemented examples of recommended activities to improve RI, adapted to their needs. Among those examples, opportunities to strengthen RI through implementing supplementary immunization activities (SIAs) were reported most frequently, including advocacy for immunization and educational activities targeted at the public and skills training targeted at health professionals. The expansion of cold chain capacity to accommodate supplies required for SIAs facilitated widening RI service delivery to reach more communities, introduce new vaccines, and reduce the risk of vaccine stock-outs. Substantial numbers of under-vaccinated children, according to the national immunization schedule, have been identified during SIAs, but it is not possible to confirm whether these children actually received missing RI doses. Micro-planning exercises for SIAs have generated data that permitted the revision of catchment populations for fixed site and outreach RI services. Some countries reported using the opportunity afforded by measles/rubella elimination to strengthen overall vaccine-preventable disease surveillance and outbreak preparedness and to introduce mandatory school-entry vaccination requirements covering other vaccines in addition to measles and rubella. Unfortunately, we were unable to obtain information regarding the cost, impact or sustainability of these activities. The evaluation of the many other strategies that have been deployed in recent years to strengthen RI systems and raise vaccination coverage was beyond the scope of this survey. We conclude by providing recommendations to encourage more countries to adapt and implement a comprehensive set of RI-strengthening activities in association with the MR elimination goal.

## 1 Background

Global experience has clearly demonstrated that, due to measles’ extraordinary contagiousness leading to herd immunity thresholds of 92–94%, measles elimination can only be achieved in the presence of near-universal vaccination coverage with two doses of measles-containing vaccine (MCV) [1], [2]. Full, equitable and sustainable routine immunization (RI) services are essential to achieve and sustain measles elimination. In countries using combined measles- and rubella-containing vaccines (MRCVs), rubella transmission should be interrupted even before measles transmission is interrupted, since rubella exhibits lesser contagiousness. The Measles-Rubella (MR) Global Strategic Plan 2012–2020 reconfirms that the achievement of regional and global MR goals requires robust and effective health and immunization systems, and that strengthening RI contributes to strengthening health systems [3]. Strategies and activities designed to achieve MR elimination offer multiple opportunities to strengthen RI service delivery, including raising vaccination coverage and closing gaps in the population immunity profile, strengthening vaccine-preventable disease (VPD) surveillance, increasing operational reach and efficiency, and rationalizing the use of resources.

The correct balance between RI service delivery and the implementation of the additional strategies required to eliminate VPDs such as measles and rubella has been debated *ad nauseam* for >50 years.

Achieving this balance is context-specific. Studies show that in countries with strong health systems, the implementation of additional strategies, particularly SIAs, causes little or no disruption to RI service delivery, but where systems are chronically weak, difficulties may arise [4]. Staff may be temporarily diverted from health facility duties, causing postponement or cancellation of routine services; funds, cold chain equipment and/or vehicles may be temporarily reassigned; in some cases, staff incentives have been paid for additional strategies, but not for RI service delivery; and issues may arise related to data standardization and quality. It is therefore critical to take full advantage of the opportunities arising from VPD elimination to avoid these difficulties and strengthen RI services.

MR vaccination coverage through RI has stagnated in recent years [5]. From 2000 to 2016, estimated coverage with the first dose of measles-containing vaccine (MCV1) increased globally from 72% to 85%, although coverage has not increased since 2009 and there is significant variability in regional coverage. During 2000–2016, the number of countries providing a second dose of measles-containing vaccine (MCV2) nationally through RI services increased from 98 (51%) to 164 (85%). Estimated global MCV2 coverage steadily increased from 15% in 2000 to 64% in 2016.

During 2016, approximately 119 million persons received MCV during 33 supplementary immunization activities (SIAs)^1^, implemented in 31 countries. Reported coverage was ≥95% in 20 of 31 (61%) SIAs, but this was only confirmed by survey in 3 countries.

Consequently, the 2015 global measles control milestones were not met. With suboptimal MCV coverage, outbreaks continued to occur among susceptible individuals, including school-aged children and young adults. A mid-term review (MTR), conducted in 2016 and subsequently endorsed by the WHO Strategic Advisory Group of Experts on Immunization (SAGE), noted that current measles elimination strategies were sound but that implementation of the strategies needed improvement [6]. The MTR concluded that the full potential of using MR elimination activities to strengthen aspects of RI service delivery had not been met. Consequently, the MTR recommended that examples showing where a focus on MR elimination has led to building of the overall immunization system should be identified.

In response, the present survey was commissioned to document specific national examples from all six WHO Regions where MR elimination activities have contributed to strengthening aspects of RI service delivery. The goal is to encourage National Immunization Programme (NIP) managers to adopt similar strategies, as appropriate for their countries, and implement selected activities to strengthen RI service delivery.

## 2 Methodology

A letter was sent to WHO and partner agency immunization staff requesting them to provide a list of countries where, in their professional opinion, the implementation of activities associated with measles/rubella elimination had strengthened RI. WHO and partner agencies identified 25 countries where suitable examples were located. The same letter was then sent to key informants in those 25 countries, with follow-up by email, telephone and Skype, in order to secure a full description of the activities and, if available, quantitative or qualitative evidence of cost, impact and sustainability.

National examples were divided into six categories of recommended opportunities to strengthen RI through MR elimination activities adapted from the WHO Global Routine Immunization Strategic Plan (GRISP) [7] (Table 1). For each category, documentation provided describing the country strategies and activities was analysed and, where possible, conclusions were drawn highlighting those strategies and activities that have demonstrated impact and appear reproducible in multiple settings.

**Table 1:** Adapted GRISP categories of activities to strengthen RI through MR elimination.

Category	Activities
1	SIAs used to identify children unvaccinated or under-vaccinated with antigens other than measles and rubella
2	SIAs used to strengthen RI in other ways, e.g. social mobilization, health care worker (HCW) refresher training, additional resources for RI (e.g. cold chain), etc.
3	MR surveillance used to strengthen other VPD surveillance, identify high-risk communities, etc.
4	MR outbreak investigation used to strengthen RI, e.g. prioritize low coverage communities for antigens other than measles and rubella
5	Adoption of MR elimination goal used to close immunity gaps with antigens other than measles and rubella, e.g. through 2YL, MCV2 or RCV introduction, school entry requirements, adult vaccination, etc.
6	Expansion of HCWs’ terms of reference specifically to include RI strengthening activities

The survey identified a convenience sample of examples of recommended RI-strengthening activities in each WHO Region, but this set of examples should not be considered representative. Therefore, inter-regional and inter-category comparisons could not be made. Further research would be required to document the global scope of RI-strengthening activities associated with MR elimination.

## 3 Results: Country examples and experiences

From the 25 countries contacted, 31 examples from 23 countries (92%) from all six WHO Regions were reported in the survey, with supporting documentation (Table 2). Despite multiple reminders, two countries did not provide examples. No examples of how SIA or other MR elimination activities that may have weakened RI were reported in the current survey.

**Table 2:** Summary of eligible country examples by WHO Region.

Region	Country	GRISP categories					
		SIAs used to identify children unvaccinated or under-vaccinated with antigens other than measles and rubella	Used to strengthen RI in other ways, e.g. social mobilization, health care worker refresher training, additional resources for RI, etc.	MR surveillance used to strengthen other VPD surveillance, identify high-risk communities, etc.	MR outbreak investigation used to strengthen RI, e.g. prioritize low coverage communities for antigens other than measles and rubella	Adoption of MR elimination goal used to close immunity gaps with antigens other than measles and rubella, e.g. through 2YL, MCV2 or RCV introduction, school entry requirements, adult vaccination, etc.	Expansion of HCWs ‘terms of reference, specifically to include RI strengthening activities
AFR	Eritrea		X				
	Ethiopia		X	X			
	Kenya		X				
	Liberia		X				
	Malawi		X				
	Nigeria		X				
	Namibia		X				
	Rwanda		X				
	Tanzania		X				

AMR	Honduras	X	X				
	Mexico	X		X			X

EMR	Pakistan	X		X			

EUR	Austria				X		
	France					X	
	Germany			X			

SEAR	India		X				
	Indonesia	X					
	Timor Leste		X				

WPR	Cambodia		X		X		
	Japan			X			
	Lao PDR	X	X				
	Malaysia	X	X	X			
	Rep Korea					X	

Total	23	6	15	5	2	2	1

### 3.1 SIAs used to identify children unvaccinated or under-vaccinated with antigens other than measles and rubella

The WHO guidelines for planning, implementing and evaluating measles and measles-rubella SIAs in each WHO Region include guidance on multiple ways to utilize the opportunity of campaigns to identify unvaccinated or under-vaccinated children in order to complete their RI schedules [8]. Since 2000, dozens of countries have included these activities while implementing SIAs. In the current survey, countries reported that children missing RI doses were identified prior to SIAs during social mobilization and house-to-house (H2H) canvassing by health extension workers, Red Cross workers or community volunteers, e.g. in Indonesia [9], Liberia [10] and Namibia [11]. In other countries, children missing RI doses were identified during SIAs by reviewing home-based records (HBRs) at the time of receiving MCV, e.g. in Pakistan (at fixed sites only) [12]. In some cases, e.g. in Honduras [13] and the Lao People’s Democratic Republic (PDR) [14], children who missed MCV during SIAs and those missing other RI doses were identified during rapid coverage monitoring (RCM) and post-SIA surveys.

Red Cross volunteers have implemented pre-SIA house-to-house canvassing activities in >100 SIAs in 46 African countries since 2000. Over 50,000 volunteers have been trained and >11 million households (HHs) visited. Red Cross workers observed that in three recent SIAs (in Kenya, Namibia and Zambia), although substantial numbers of children missing RI doses were identified, little information was available to confirm whether these children did in fact receive the vaccine doses they needed [15]. In the absence of systematic follow-up, it is not known what proportion of these children were eventually vaccinated. It was proposed several years ago to include resources when planning and budgeting SIAs to permit the follow-up of children identified as requiring additional RI vaccine doses and monitor compliance [16]. However, during the present survey of 23 countries, no national reports were received confirming that this recommendation was put into practice with the exception of MR SIAs in Malaysia in 2017, where arrangements were made to reschedule children identified as having missed RI doses during 2016–17 [17].

As in many Member States in the WHO Americas Region, MRCV coverage in Mexico is high enough to permit suspension of the SIA strategy. However, National Health Weeks are conducted three times per year to supplement RI and sustain high coverage. In the course of H2H visits, rapid coverage monitoring (RCM) assessments are conducted and HBRs are routinely screened in order to identify children who missed MRCV and other RI doses [18].

### 3.2 SIAs used to strengthen RI in other ways

WHO guidelines also recommend using the opportunity of SIAs to expand cold chain capacity and transport fleets, to extend public advocacy and education for RI through social mobilization and house-to-house (H2H) canvassing, and to give refresher training of healthcare workers (HCWs) to implement SIAs and consolidate RI knowledge and skills including vaccine supply and management, vaccination technique, waste disposal, investigation and reporting of adverse events following immunization (AEFIs), VPD surveillance, risk assessments, data management and transmission, and more.

In this survey, the most common activity associated with SIAs was the provision of refresher training to HCWs on a wide variety of RI knowledge and skills. This was confirmed by key informants in all WHO Regions, e.g. in AFR (Tanzania, 2011; Malawi, 2015; Eritrea, 2015; Kenya, 2016; Nigeria 2017–18), in AMR (Honduras, 2016), in EMR (Pakistan 2014–15), in EUR (Azerbaijan, 2014; Georgia, 2014; Kyrgyzstan, 2015), in SEAR (Indonesia, 2016–17; Timor Leste, 2015 [19]) and in WPR (Cambodia, 2013 and 2017; Lao PDR, 2017; Malaysia, 2017).

The expansion of cold chain capacity using SIA funds during preparations for SIAs was also mentioned frequently. Additional equipment was budgeted and procured in advance of SIAs, e.g. in AFR (Malawi, 2015), in AMR (Honduras, 2016) and in SEAR (Timor Leste, 2015). Prior to SIAs in Tanzania in 2014, an additional 240 refrigerators and 10,000 vaccine carriers were procured. In Pakistan during the 2014–15 SIAs, an additional 20,000 standard vaccine carriers and 800 cold boxes significantly increased programme capacity in RI service delivery [20]. In Liberia in 2015, a national cold chain assessment was conducted prior to the SIA and teams were deployed to repair equipment as needed. A logistical supply assessment tool was also used to assess pre-SIAs preparedness. Additional cold boxes and vaccine carriers were procured to strengthen cold chain capacity nationwide. In Rwanda in 2013, SIAs funds were allocated to replace part of the kerosene-fuelled refrigerator stock with solar-powered refrigerators [21]. In Ethiopia in 2016, 40% of measles SIAs operational funding was used to purchase cold chain equipment [22].

Preparations for SIAs have facilitated the development of micro-plans to more accurately estimate target populations and to validate denominators for monitoring RI performance. In Tanzania in 2011, SIAs micro-plans were used to identify new sites for outreach and mobile services employing the Reaching Every Child approach [23]. In Nigeria in 2017–18, the enumeration of HHs using geographic information system technology, which was initially employed to validate denominators for children 9–59 months of age for SIAs micro-planning, was then used to validate denominators for infants 0–11 months of age for improved RI coverage monitoring [24]. Similarly, micro-planning exercises for measles SIAs in Liberia in 2015 generated data that permitted the revision of catchment populations for use in future RI fixed site and outreach micro-planning.

### 3.3 MR surveillance used to strengthen other VPD surveillance

Almost all countries have established case-based measles surveillance and monitor WHO-recommended indicators to achieve measles elimination. Many countries have also established case-based rubella surveillance. In most cases, MR surveillance and reporting is integrated into the national disease surveillance system. Some countries have also included the theory and practice of VPD surveillance in the refresher training provided to HCWs and other cadres during preparations for SIAs, e.g. in Ethiopia in 2016 and Pakistan in 2014–15. During pre-SIA preparations, orientation for HCWs and other cadres to consolidate RI knowledge and skills frequently included detailed training on AEFI surveillance including the identification, treatment and reporting of and response to suspected AEFI cases.

In addition, some countries have used the opportunity afforded by the establishment of case-based measles or rubella surveillance to strengthen VPD surveillance in general. When measles case-based surveillance was introduced in Pakistan in 2009, reporting tools were modified to record additional epidemiological data on VPD cases including vaccination status and laboratory results, monthly reporting was replaced by weekly reporting, and measles surveillance indicators were extended to all VPDs [12].

To comply with WHO European Regional guidance on MR elimination, the Federal Government of Germany updated legislation in 2014 to introduce nationwide case-based rubella surveillance. At the same time, four more VPDs (rubella, mumps, pertussis and varicella) were made notifiable and subject to case investigation and response [25]. Several targeted communication activities to inform public health authorities and the professional community helped to strengthen the wider disease surveillance system and increase awareness about rubella and rubella vaccination.

In Malaysia, the prevention of nosocomial measles transmission led to revision of hospital infection control guidelines requiring vaccination with MMR and other selected RI antigens (hepatitis B, influenza and typhoid) for all staff involved in patient care, food handling and laboratory work [26]. Furthermore, measles risk assessment methodology was extended to cover other VPDs.

In Mexico, the active search for measles and rubella cases in municipalities reporting inadequate MR surveillance sensitivity, especially those located along national borders, those with substantial tourist arrivals, and those with low RI coverage, was extended to include all VPDs [18].

### 3.4 MR outbreak investigation used to strengthen RI

Following the adoption of the MR elimination goal, Cambodia conducted a nationwide series of professional training exercises on measles outbreak preparedness, investigation and response. These courses also served to build capacity for preparedness and response to other VPD and non-VPD outbreaks [27]. Furthermore, in the course of measles outbreak responses, vaccinators check HBRs and vaccinate children who are missing doses of other RI antigens.

In Austria, slow progress towards verifying MR elimination provoked the government to implement a series of advocacy and multi-media promotional activities to boost awareness and demand for RI services among health care professionals and the general public. This included strengthening knowledge and skills related to VPD surveillance and outbreak control, particularly related to timeliness and data quality [28]. Using outbreak investigations including contact tracing and data analysis as training opportunities, capacity building has had a positive impact on the control of other infectious diseases.

### 3.5 Adoption of MR elimination goal used to close immunity gaps with other antigens

In France, slow progress towards verifying MR elimination caused primarily by inadequate MCV population immunity provoked the government to enact legislation in 2017 mandating school-entry vaccination for 11 antigens including MRCV [29]. This legislation came into force at the beginning of 2018 and is anticipated to have a positive impact on RI coverage nationwide.

Similarly, in the Republic of Korea, after the school entry vaccination requirement was extended to include MCV2, legislation was modified twice to include proof of vaccination with all RI antigens [30]. Although vaccination is technically voluntary, public education aims to explain the balance between individual choice and the social duty to prevent community spread of VPDs. As a result, this measure has had a positive impact on RI coverage nationwide. In 2013, coverage with MCV2, IPV4 and DTP5 vaccine was >95% among children at school entry, having risen from around 80% a decade earlier.

### 3.6 Expansion of HCWs’ ToRs specifically to include RI strengthening activities

Government policy in Mexico provides for comprehensive Well Child visits every 2 months, up to the 5th birthday, which require, among other things, that HCWs review every infant’s and child’s immunization status at each visit and complete the RI schedule as necessary [18].

## 4 Discussion and conclusions

A robust RI system should assure that all children have access to recommended vaccines; all HCWs are fully trained to determine eligibility for a given immunization and how to administer the vaccine; maintains a record keeping system that permits easy identification of under-vaccinated children at each healthcare contact; deploys a system to remind parents when vaccinations are due or past due; includes educational programmes and outreach to communities to maximize demand for immunization; implements a sensitive surveillance system that detects VPDs and determines the epidemiology and burden of each VPD; determines whether the cases are a result of failure to vaccinate or vaccine failure; and links immunization to provision of other critical health services, such as growth monitoring.

Although the strategies and activities defined in the Global MR Strategic Plan 2012–20 for achieving MR elimination include multiple opportunities to strengthen RI systems, the mid-term review (MTR) of progress conducted in 2016 concluded that the full potential of these opportunities had not been met. The MTR noted that further investigation is indicated to better understand the reasons why countries have not always used MR elimination activities to strengthen RI and what steps could be taken to ensure more widespread implementation in the future. The present survey constituted a first step in that direction and revealed that countries in all six WHO Regions have implemented many, but not all, of the recommended activities, adapted to their needs.

This survey was designed to solicit positive examples of how a focus on MR elimination could improve the overall routine immunization system. Where RI service delivery is weak, MR elimination activities may have negative impact on the RI system and, going forward, this will be very important to determine to avoid such outcomes. However, the goal of this survey was to find positive examples in hopes of stimulating persons engaged in enhancing activities to reach the regional MR elimination goals to consider adopting similar procedures to strengthen RI and to show that undertaking major interventions, such as SIAs, could have a positive impact on RI.

Preparing for SIAs has facilitated a wide range of RI advocacy and educational activities targeted at the public and health professionals, through widespread social mobilization, H2H canvassing and multiple media channels. Furthermore, additional funds have been deployed to expand cold chain capacity to accommodate extra vaccine supplies required for the surge in vaccination over a short period. These additional resources are extremely valuable for expanding RI service delivery to reach more communities, for introducing new vaccines, and for reducing the risk of vaccine stock-outs.

In certain lower-income countries (LICs), micro-planning exercises for measles SIAs generated data that permitted the revision of catchment populations in order to fine-tune micro-planning for fixed site and outreach RI services.

Furthermore, during SIAs in many countries substantial numbers of under-vaccinated children were identified, but it is not possible to confirm whether these children actually received missing RI doses. Although it was proposed several years ago to earmark specific resources when planning and budgeting SIAs to ensure that children requiring additional RI vaccine doses were followed up and that compliance was monitored [16], no examples where this specific recommendation was implemented were identified in the present survey.

High-income countries (HICs), where SIAs have never been implemented or have not been implemented recently, did not report interventions related to SIAs. In many HICs, other RI-strengthening activities have been implemented in association with the pursuit of MR elimination, especially public advocacy and awareness-raising activities, often in association with National Immunization Week initiatives.

Almost all countries have now established case-based measles or MR surveillance and monitor standard indicators in accordance with WHO recommendations. However, some countries have used the opportunity to strengthen VPD surveillance in general. A few countries, mainly HICs, reported enhancing case-based surveillance and outbreak preparedness for VPDs in addition to measles and rubella, legislating mandatory school-entry vaccination requirements with vaccines in addition to measles and rubella, and ensuring that during Well Child clinics HCWs always review infants’ and children’s vaccination status and complete the RI schedule as appropriate. However, it appears that mandatory school-entry vaccination requirements have not yet been introduced and enforced in enough countries, especially large LICs and MICs, to make a significant global impact.

WHO and partner agencies have advocated strongly with countries to establish second-year-of-life (2YL) platforms to expand infant immunization beyond the first birthday and, taking advantage of the introduction of the MCV2/MRCV2 dose, to raise awareness of and compliance with all booster doses included in the second year of the national immunization schedule [31]. However, in the countries contacted in this survey, no examples of 2YL platforms were identified.

Unfortunately, despite requests for data, we were unable to obtain information regarding the cost, impact or sustainability of the activities. With few exceptions, budgets for SIAs do not itemize expenditure on RI-strengthening activities. Furthermore, quantitative data to monitor impact or the overall sustainability of these measures have not been collected. This is an area which will need to be addressed more aggressively in the future, in order to demonstrate value and encourage more countries to adapt and implement a comprehensive set of RI-strengthening activities in association with the MR elimination goal.

The focus of this study was on using the opportunity resulting from the pursuit of regional MR elimination goals to strengthen RI. It provided a number of examples of how efforts focused on MR elimination have helped strengthen RI. Our objective was to document examples of practices that other NIP managers can adapt and implement but we were unable to rank them because their respective impact was not quantified. The study shows that we must hold stakeholders at all levels accountable not only for achieving MR elimination but also taking advantage of those activities to strengthen RI sustainably. However, we fully recognize that the establishment and maintenance of strong RI systems clearly require a wider range of measures than those associated with MR elimination.

## 5 Recommendations

a. A protocol, including a user-friendly checklist, should be developed to uniformly assess the impact of SIAs on RI and other aspects of national immunization systems so that those responsible for planning SIAs will understand how they will be evaluated.
b. National immunization programmes should document if an activity related to MR elimination efforts negatively impacts RI service delivery and document such information to help in determining how to avoid such results in the future.
c. Current efforts to strengthen RI through SIAs should be sustained and, where possible, expanded. WHO Regional post-SIA technical report templates should be revised to:
(i) include quantitative indicators to monitor RI-strengthening activities, especially those related to identifying children with incomplete RI schedules and following them up in a timely fashion to ensure that their schedules are completed, and
(ii) collect quantitative and qualitative information on the cost, impact and sustainability of RI-strengthening activities.
d. All SIAs (MR, cholera, Japanese encephalitis, meningitis, yellow fever, etc.) offer the opportunity to review HBRs and identify unvaccinated and under-vaccinated children. However, specific resources should be included in SIA budgets to ensure that these children are followed up to complete their RI schedules.
e. Effective steps to close immunity gaps in the population should be promoted, including requiring school-entry and, where appropriate, other vaccination checks, especially in LICs and MICs with large populations, to increase global impact.
f. After finalizing technical guidelines and raising adequate funding, efforts to introduce 2YL platforms, taking advantage of MCV2/MRCV2 introduction to raise awareness of and compliance with RI booster doses scheduled in the second year of life, should be accelerated.

## Conflicts of interest

None.

## Ethical guidelines

Manuscript was prepared from country reports, no personal data were accessed, no invasive procedures were performed, and therefore no ethics clearance was required.

## Footnotes

1 Supplemental immunization activities (SIAs) generally are carried out using 2 target age ranges. An initial, nationwide catch-up SIA focuses on all children aged 9 months–14 years, usually regardless of prior vaccination status, with the goal of eliminating susceptibility to measles in the general population. Periodic follow-up SIAs then focus on all children born since the last SIA. Follow-up SIAs generally are conducted nationwide every 2–4 years and focus on children aged 9–59 months; their goal is to eliminate any measles susceptibility that has developed in recent birth cohorts and to protect children who did not respond to MCV1.
